# Regulating amyloidogenesis through the natural triggering receptor expressed in myeloid/microglial cells 2 (TREM2)

**DOI:** 10.3389/fncel.2014.00094

**Published:** 2014-03-31

**Authors:** Brandon M. Jones, Surjyadipta Bhattacharjee, Prerna Dua, James M. Hill, Yuhai Zhao, Walter J. Lukiw

**Affiliations:** ^1^LSU Neuroscience Center, Louisiana State University Health Sciences Center (LSUHSC)New Orleans, LA, USA; ^2^Department of Health Information Management, Louisiana State UniversityRuston, LA, USA; ^3^Department of Ophthalmology, Health Sciences Center, Louisiana State UniversityNew Orleans, LA, USA; ^4^Department of Neurology, Health Sciences Center, Louisiana State UniversityNew Orleans, LA, USA

**Keywords:** Alzheimer's disease (AD), amyloidogenesis, miRNA-34a, TREM2, microglial cells, inflammation, phagocytosis, Aß42 peptides

Amyloidogenesis, the progressive accumulation of amyloid-beta (Aβ) peptides into insoluble, toxic, senile plaque lesions is one of the major defining features of the Alzheimer's disease (AD) brain. Normally, Aβ42 peptides are cleared from the extracellular space by natural phagocytic mechanisms, but when this intrinsic sensing and clearance system is functionally compromised or defective, Aβ42 peptides accumulate. Largely due to their intensely hydrophobic character, under physiological conditions Aβ42 peptides have a strong tendency to self-aggregate into higher order neurotoxic and pro-inflammatory fibrillar aggregates. While the Aβ peptide-clearing mechanisms are highly complex, one particularly important molecular sensor for Aβ42 peptide clearance is the triggering receptor expressed in myeloid/microglial cells 2 (TREM2; encoded at chr6p21.1), a variably glycosylated 230 amino acid transmembrane spanning stimulatory receptor of the immunoglobulin/lectin-like gene superfamily strongly associated with innate-immune, pro-inflammatory, and neurodegenerative signaling in AD. TREM2 is highly and almost exclusively expressed on the outer plasma membrane of microglial cells, the resident phagocytic and scavenging neuroimmune macrophages of the human central nervous system (CNS). As AD progresses, microglia appear to become progressively dysfunctional; TREM2 becomes down-regulated and microglia lose their ability to clear Aβ42 peptides while producing and releasing neurotoxins, reactive oxygen species (ROS), and pro-inflammatory cytokines that further promote Aβ42 production and pathological aggregation (Schmid et al., 2002; Alexandrov et al., 2013; Boutajangout and Wisniewski, 2013; Hickman and El Khoury, 2013).

**Figure 1:**
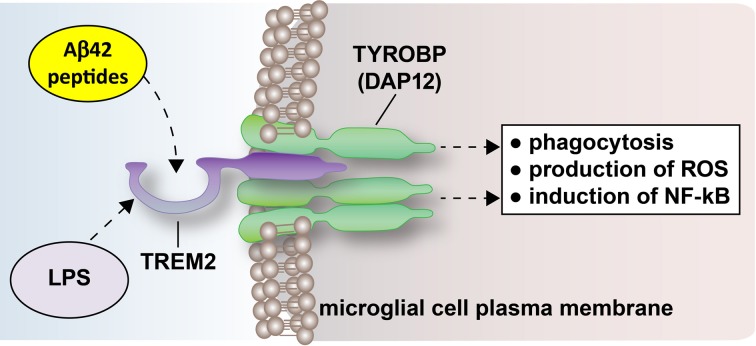
**The triggering receptor expressed in myeloid/microglial cells type 2 (TREM2) is a 230 amino acid, ~25.4 kDa integral trans-membrane glycoprotein sensor spanning the lipid bilayer of CNS microglial cells**. Very recently, TREM2 has been shown to act as a phagocytic receptor of bacterial lipopolysaccharide (LPS), Aβ42 peptides and other cellular end-stage noxious cellular products (N'Diaye et al., 2009; Guerreiro and Hardy, 2013; Jonsson et al., 2013). TREM2-LPS or TREM2-Aβ peptide recognition may be achieved in part through a pathogen-associated molecular pattern (PAMP) characteristic of highly specific molecular features located on LPS or Aβ42 molecules (Boutajangout and Wisniewski, 2013; Zhao et al., 2013; unpublished observations). Transmembrane signaling via TREM2 is in part accomplished through a trans-membrane adapter, tyrosine kinase binding protein called TYROBP, also known as the DNAX-activation protein 12 (DAP12) or the polycystic lipomembranous osteodysplasia with sclerosing leukoencephalopathy (PLOSL) protein (Schmid et al., 2002; Jonsson et al., 2013). TREM2 signaling triggers the phagocytic uptake of cellular debris and is associated with the further down-stream induction of reactive oxygen species (ROS) and the pro-inflammatory transcription factor NF-kB (Charles et al., 2008; N'Diaye et al., 2009). Up-regulation of ROS and NF-kB are a characteristic feature of inflammatory neurodegeneration and increasing Aβ42 peptide load in AD brain (Hickman and El Khoury, 2013; Jonsson et al., 2013; Zhao et al., 2013). Interestingly, TREM2 expression is critical for the clearance of neural debris of the injured or lesioned CNS, and loss-of-function mutations in TREM2 or TYROBP (DAP12) are linked to presenile dementias characteristic of ALS or AD-type neocortical degeneration (Charles et al., 2008; Guerreiro and Hardy, 2013; Cady et al., 2014; Jiang et al., 2014). This highly schematicized figure was adapted in part from Nimmerjahn et al. (2005), Neumann and Takahashi (2007), Guerreiro and Hardy (2013), and Neumann and Daly (2013).

Recently, much interest in the molecular biology, genetics, and epigenetics of TREM2 expression, and its potential for sensing and scavenging Aβ42 peptides in AD and other progressive neurodegenerative diseases has arisen. TREM2's critical importance is underscored by seven recent observations: (1) that relatively rare mutations of TREM2 (or of its coupling protein, DAP12, also known as TYROBP; see Figure 1) are currently associated with the progressive, pre-senile dementing illnesses Nasu-Hakola syndrome, polycystic lipomembranous osteodysplasia with sclerosing leucoencephalopathy (POSL), sporadic amyotrophic lateral sclerosis (ALS), and AD (Nimmerjahn et al., 2005; Neumann and Takahashi, 2007; Guerreiro and Hardy, 2013; Zhao and Lukiw, 2013; Zhao et al., 2013; Cady et al., 2014); (2) that down-regulation in the phagocytic ability of microglia to degrade Aβ42 peptides in AD, and down-regulation in TREM2 expression, has been reported in sporadic AD brain tissues (Hickman and El Khoury, 2013; Zhao et al., 2013); (3) that TREM2 knockdown has been shown to exacerbate age-related neuro-inflammation and enhance cognitive deficiency in senescence accelerated mouse prone 8 (SAMP8) mice (Jiang et al., 2014); (4) that microglial TREM2 gene expression in cell culture, both at the level of mRNA and protein, have been shown to be remarkably sensitive to external cytokine stressors such as tumor necrosis factor alpha (TNFα; a pro-inflammatory adipokine known to be up-regulated in AD brain; Zhao et al., 2013; unpublished observations); (5) that AD-relevant pro-inflammatory neurotoxins such as bacterial lipopolysaccharide (LPS) and environmentally abundant toxic metals such as aluminum strongly down-regulate TREM2 and the ability of microglial cells to phagocytose extracellular debris (Hickman and El Khoury, 2013; Zhao et al., 2013; unpublished observations); (6) that down-regulation in the expression of TREM2 appears to be regulated in part by the up-regulation of the microglial-enriched, NF-kB-sensitive microRNA-34a (miRNA-34a), and perhaps other NF-kB-sensitive miRNAs, and (7) that both anti-NF-kB and anti-microRNA (AM-RNA) strategies have been shown to be useful in the restoration of homeostatic TREM2 gene expression levels and the neutralization of pro-inflammatory signaling and amyloidogenesis, at least *in vitro* (Hill et al., 2009; Pogue et al., 2009, 2010; Alexandrov et al., 2012, 2013; Zhao et al., 2013; unpublished observations). From what we know so far it is tempting to speculate that (1) loss-of function of TREM2 due to genetic mutations in familial AD may have the same end effects on phagocytosis as down-regulation of a fully functional TREM2 in sporadic AD; and that (2) modest TREM2 over-expression might be useful in enhancing the scavenging and removal of cellular debris in the CNS, including neurotoxic and self-aggregating Aβ42 peptides. Importantly, TREM2 signaling has been recently shown to be selectively inducible and manipulated from outside of the cell, suggesting that the modulation of TREM2 expression may be effectively regulated using highly specific targeting via drug-based pharmacological strategies exogenously supplied (Alexandrov et al., 2013; Lukiw, 2013; Zhao et al., 2013).

AD represents a highly complex, insidious, progressive, multi-factorial brain dysfunction whose incidence is reaching epidemic proportions. Despite the billions of dollars already spent on AD research, including multiple Aβ immunization and immunotherapy strategies, there is still no adequate treatment or cure for AD, and the development and implementation of novel, more effective treatment strategies are critical. Recruitment and harvesting of the TREM2 mechanism as a potent, endogenous Aβ42-peptide scavenging activity may represent a singularly attractive new direction for the clinical management of AD. Indeed, as a natural sensor and scavenger of noxious cellular debris TREM-2 stimulation may turn out (1) to be remarkably neuroprotective against both amyloidogenesis and age-related neuro-inflammation; while (2) significantly reducing the progressive cognitive impairment associated with amyloidogenesis and inflammatory neurodegeneration in the CNS (Hickman and El Khoury, 2013; Zhao et al., 2013; Cady et al., 2014; Jiang et al., 2014). Put another way, a decline in TREM2's contribution to the innate immune response, in part driving amyloid-clearance deficits and progressive degeneration characteristic of the AD process, suggest novel therapeutic targets and treatment strategies directed at maintaining natural and homeostatic TREM2 functions. This may be accomplished not only through the direct stimulation of TREM2 itself but also through the poorly understood downstream TREM2-linked TYROBP (DAP12) signaling pathways responsible for; (1) the actual phagocytosis of extracellular molecules; (2) the production of damaging quantities of ROS; (3) the induction of pro-inflammatory signals via NF-kB; and (4) the maintenance of homeostatic microglial function that together may diminish amyloidogenesis and the intercellular propagation of pathogenic signaling in the AD affected brain (Boutajangout and Wisniewski, 2013; Guerreiro and Hardy, 2013; Zhao and Lukiw, 2013; Jiang et al., 2014; Figure 1).
